# Equilibria in cobalt(II)–amino acid–imidazole system under oxygen-free conditions: effect of side groups on mixed-ligand systems with selected L-α-amino acids

**DOI:** 10.1186/s13065-016-0160-5

**Published:** 2016-03-31

**Authors:** Magdalena Woźniczka, Andrzej Vogt, Aleksander Kufelnicki

**Affiliations:** Department of Physical and Biocoordination Chemistry, Faculty of Pharmacy, Medical University of Łódź, Muszyńskiego 1, 90-151 Łódź, Poland; Faculty of Chemistry, University of Wrocław, F. Joliot-Curie 14, 50-383 Wrocław, Poland

**Keywords:** Cobalt(II), L-α-Amino acid, Imidazole, Oxygen-free ternary complexes

## Abstract

**Background:**

Heteroligand Co(II) complexes involving imidazole and selected bio-relevant L-α-amino acids of four different groups (aspartic acid, lysine, histidine and asparagine) were formed by using a polymeric, pseudo-tetrahedral, semi-conductive Co(II) complex with imidazole–[Co(imid)_2_]_n_ as starting material. The coordination mode in the heteroligand complexes was unified to one imidazole in the axial position and one or two amino acid moieties in the appropriate remaining positions. The corresponding equilibrium models in aqueous solutions were fully correlated with the mass and charge balance equations, without any of the simplified assumptions used in earlier studies. Precise knowledge of equilibria under oxygen-free conditions would enable evaluation of the reversible oxygen uptake in the same Co(II)–amino acid–imidazole systems, which are known models of artificial blood-substituting agents.

**Results:**

Heteroligand complexes were formed as a result of proton exchange between the two imidazole molecules found in the [Co(imid)_2_]_n_ polymer and two functional groups of the amino acid. Potentiometric titrations were confirmed by UV/Vis titrations of the respective combinations of amino acids and Co-imidazole. Formation of MLL′ and ML_2_L′ species was confirmed for asparagine and aspartic acid. For the two remaining amino acids, the accepted equilibrium models had to include species protonated at the side-chain amine group (as in the case of lysine: MLL′H, ML_2_L′H_2_, ML_2_L′H) or at the imidazole N1 (as in the case of histidine: MLL′H and two isomeric forms of ML_2_L′). Moreover, the Δlog_10_ *β*, log_10_ *β*_stat_, Δlog_10_ *K*, and log_10_ *X* parameters were used to compare the stability of the heteroligand complexes with their respective binary species. The large differences between the constant for the mixed-ligand complex and the constant based on statistical data Δlog_10_*β* indicate that the heteroligand species are more stable than the binary ones. The parameter Δlog_10_*K*, which describes the influence of the bonded primary ligand in the binary complex Co^II^(Himid) towards an incoming secondary ligand (L) forming a heteroligand complex, was negative for all the Amac ligands (except for histidine, which shows stacking interactions). This indicates that the mixed-ligand systems are less stable than the binary complexes with one molecule of imidazole or one molecule of amino acid, in contrast to Δlog_10_ *β*, which deals with binary complexes Co^II^(Himid)_2_ and Co^II^(AmacH_−1_)_2_ containing two ligand molecules. The high positive values of the log_10_*X* disproportionation parameter were in good agreement with the results of the Δlog_10_*β* calculations mentioned above.

**Conclusion:**

The mixed-ligand MLL′-type complexes are formed at pH values above 4–6 (depending on the amino acid used), however, the so-called “active” ML_2_L′-type complexes, present in the equilibrium mixture and known to be capable of reversible dioxygen uptake, attain maximum share at a pH around nine. For all the amino acids involved, the greater the excess of amino acid, the lower the pH where the given heteroligand complex attains maximum share. The results of our equilibrium studies make it possible to evaluate the oxygenation constants in full accordance with the distribution of species in solution. Such calculations are needed to drive further investigations of artificial blood-substituting systems.

## Background

Heteroligand Co(II)–L-α-amino acid–imidazole complexes are formed with protein amino acids in accessible coordination sites under an oxygen-free atmosphere [1]. Those paramagnetic, high-spin, mixed-ligand complexes of Co(II) contain six coordination sites. The structure is regarded as analogous to the binary amino acid complexes of Co(II) and other divalent metals, where the amino acid chelate rings are known to be in an equatorial *trans*-position [2]. The axial sites are occupied by imidazole (coordinated by N3) and a water molecule [3]. Due to the “trans-effect” of imidazole, these complexes are capable of the multiple cyclic uptake and release of molecular oxygen and therefore, are capable of imitating natural O_2_ carriers. In addition they exhibit a suitable temperature range (0–40 °C) for a full equilibrium displacement to the left or right, and are formed from ligands which are non-volatile and low-toxic. It is important to note that in order to obtain the heteroligand complex, a solid, semi-conductive polymeric complex [Co(imid)_2_]_n_ is used as starting material to ensure the position of imidazole in one of the axial sites [4].

Existing literature data suggests that heteroligand complexes in the cobalt(II)–amino acid–imidazole systems are formed within the range pH 6–10 [1, 3, 5]. This indicates the deciding donor properties of the amino groups: they dissociate in basic medium. The structures of the mixed-ligand complexes have been confirmed *inter alia* by the molar neutralization coefficient of imidazole [5] released from the inner coordination sphere of the heteroligand complex, and by additional results obtained in the presence of O_2_ [6]. As one of the two imidazole molecules is known to be released to solution from the Co(imid)_2_ unit during formation of the mixed-ligand complex, it may be assumed that two amino acid ligands are coordinated via the amino group nitrogens and hydroxyl oxygens of the carboxyl groups. A water molecule or an OH^−^ group may be expected as the remaining sixth donor. Earlier experiments carried out with analogous systems [6] suggest that the uptake of dioxygen does not change the pH, which would undoubtedly occur if O_2_ replaced a hydroxyl group. Therefore, the remaining sixth donor is evidently the oxygen of a water molecule. On the other hand, an alternative heteroligand complex, though inactive towards dioxygen uptake, may involve only one amino acid in the equatorial plane but three water molecules in the remaining sites.

The stability constants of mixed-ligand cobalt(II) complexes, with amino acids as primary ligands and imidazole as secondary ligand, under oxygen-free conditions have so far been determined potentiometrically for glycine, DL-α-alanine and  DL-valine [7], but the stability constants resulting from combined potentiometric and spectrophotometric titrations have been determined only for L-α-alanine (a monoaminocarboxylic acid) [8]. It should be emphasized that coordinating interactions in the cobalt(II)–amino acid–imidazole systems have been also investigated in solid state: with acetyl- DL-phenylglycine [9], N-acetyl,  N-benzoyl and  N-tosyl derivatives of amino acids [10] as well as in solution: with imidazole-4-acetic acid [11], bis(imidazolyl) derivatives of amino acids [12], 1,2-disubstituted derivative of L-histidine [13] and biomimetic models of coenzyme B_12_ [14].

**Fig. 1:**
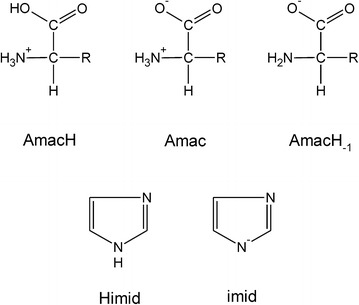
Abbreviations used for naming the ligand forms

In the present work, the investigations have been extended from our previous studies with L-α-alanine [8] to a number of amino acids representative of the other four groups: monoaminodicarboxylic acids (L-α-aspartic acid, Asp), diaminomonocarboxylic acids (L-α-lysine, Lys), amino acids with a heterocyclic ring (L-α-histidine, His), as well as amino acids with an amide side-chain group (L-α-asparagine, Asn). The forms of the ligands under study were specified by abbreviated names (Fig. 1). Prior to the experiments with these heteroligand systems, similar experiments using solutions of binary parent species had been performed under the same conditions by both methods used in the present study: pH-potentiometry and UV/Vis spectrophotometry. The essential value of determining the formation constants of the heteroligand species is that the procedure allows the stability constants, *K*_O2_, of the corresponding Co(II)—dioxygen complexes to be evaluated based on the full mass balance equations without any simplifying assumptions.

## Results and discussion

Heteroligand complexes are formed as a result of proton exchange between the two imidazole molecules found in the [Co(imid)_2_]_n_ polymer and two functional groups of the amino acid [8]. Accurate protonation constants of the amino acids and imidazole (Table 1) and formation constants of the binary complexes are needed to determine the formation constants of heteroligand complexes in reactions (1) and (2), which are also given in Table 1. Under more acidic conditions, the predominating reaction is: (charges omitted for clarity)

1 $${\rm Co{(imid)_2}}\,+\, {\rm AmacH} \, =\, \mathop{{\rm Co(AmacH}_{{-}1}){\rm Himid}} \limits_{{\rm MLL}^\prime} \,+\, {\rm Himid}$$

Then, along with alkalization, the predominating reaction is: (charges omitted for clarity)

2 $${\rm Co{(imid)_2}}\,+\, {\rm 2\, Amac} \, =\, \mathop{{\rm Co}({\rm AmacH}_{{-}1})_2{\rm Himid}}\limits_{{\rm ML_2L}^\prime}\,+\, {\rm Himid}$$

**Table 1:** Logarithms of overall formation constants in the Co^II^(Himid)(L-α-Amac)_n_H_2_O system and UV–Vis parameters

System	*m*	*l*	*l′*	*h*	Refinement results (log_10_ *β*_*mll′h*_)	*σ* ^b^	*λ*_max_ (*ε*) nm (L mol^−1^ cm^−1^)
Co(H_2_O)_6_^2+ a^	1	0	0	−1	−8.45(3)		512 (5)
Imidazole^a^	0	0	1	1	7.28(1)	2.89	
	1	0	1	0	2.82(2)	1.49	514 (6)
	1	0	2	0	4.94(2)		506 (16)
	1	0	3	0	6.76(9)		491 (38)
	1	0	4	0	8.29(13)		
	1	0	5	0	9.68(14)		
Alanine^a^	0	1	0	1	9.75(1)	4.59	
	0	1	0	2	12.13(1)		
	1	1	0	0	4.20(1)	5.70	508 (8)
	1	2	0	0	7.65(1)		491 (11)
	1	3	0	0	9.92(1)		500 (19)
	1	1	1	0	6.97(2)	1.32	505 (15)
	1	2	1	0	9.94(4)		495 (20)
Asparagine	0	1	0	1	8.66(1)	5.99	
	0	1	0	2	10.96 (1)		
	1	1	0	0	4.25(1)	3.47	507 (8)
	1	2	0	0	7.67(1)		485 (10)
	1	3	0	0	9.23(1)		500 (17)
	1	1	1	0	6.89(1)	2.17	503 (20)
	1	2	1	0	9.70(1)		491 (22)

Temp. 25.0 ± 0.1 °C, *I* = 0.5 mol L^−1^ (KNO_3_). Programs: Hyperquad 2008 and HypSpec. Standard deviations at the last decimal points—in parentheses. *β*_*mll’h*_ = [M_*m*_L_*l*_L′_*l′*_H_*h*_]/[M]^*m*^[L]^*l*^[L′]^*l′*^[H]^*h*^, where M = Co(II), L = AmacH_-1_, L′ = Himid, H = proton

^a^ Results for Co(H_2_O)_6_^2+^, Ala and Imidazole taken from previous paper [8]

^b^ *σ* statistical residual parameter of Hyperquad [27]

### L-α-Asparagine (Asn)

**Fig. 2:**
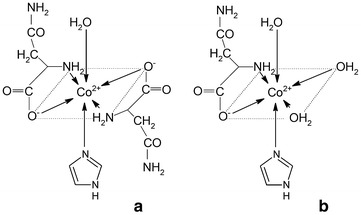
Suggested coordination modes of the ternary Co(II)–Himid–L-α-Asn complexes: **a** ML_2_L′; **b** MLL′

**Fig. 3:**
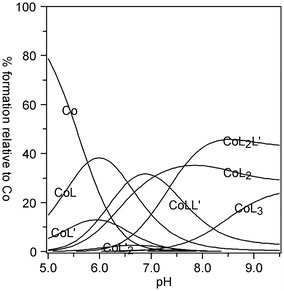
Distribution diagram of complex species versus pH for a solution of Co[(imid)_2_]_n_ and asparagine in molar ratio 1:5. *C*_Co_ = 0.01 mol L^−1^. L–asparagine (AsnH_-1_), L′–imidazole (Himid)

For the system with L-α-asparagine, two M/L/L′/H ratios have been suggested (Fig. 2). The exact coordination modes were assumed from previous literature reports and evidenced by successful refinement of the convergence between the experimental and theoretical titration curves, as well as by Vis spectroscopy. In both the heteroligand structures (ML_2_L′ and MLL′) chelation only occurs due to the carboxyl and amino groups at the α-carbon (Fig. 2). It is known that above pH 13, asparagine is a potentially tridentate ligand [15]. In such an alkaline medium, the amide-NH_2_ side group is deprotonated, which may lead to other coordination modes. However, within the pH range 9–10, used in the present study, asparagine behaves only as a bidentate ligand, in a similar way to alanine, among other amino acids [8]. The relevant determined stability constants and speciation diagram are presented in Table 1 and Fig. 3.

### L-α-Aspartic acid (Asp)

**Fig. 4:**
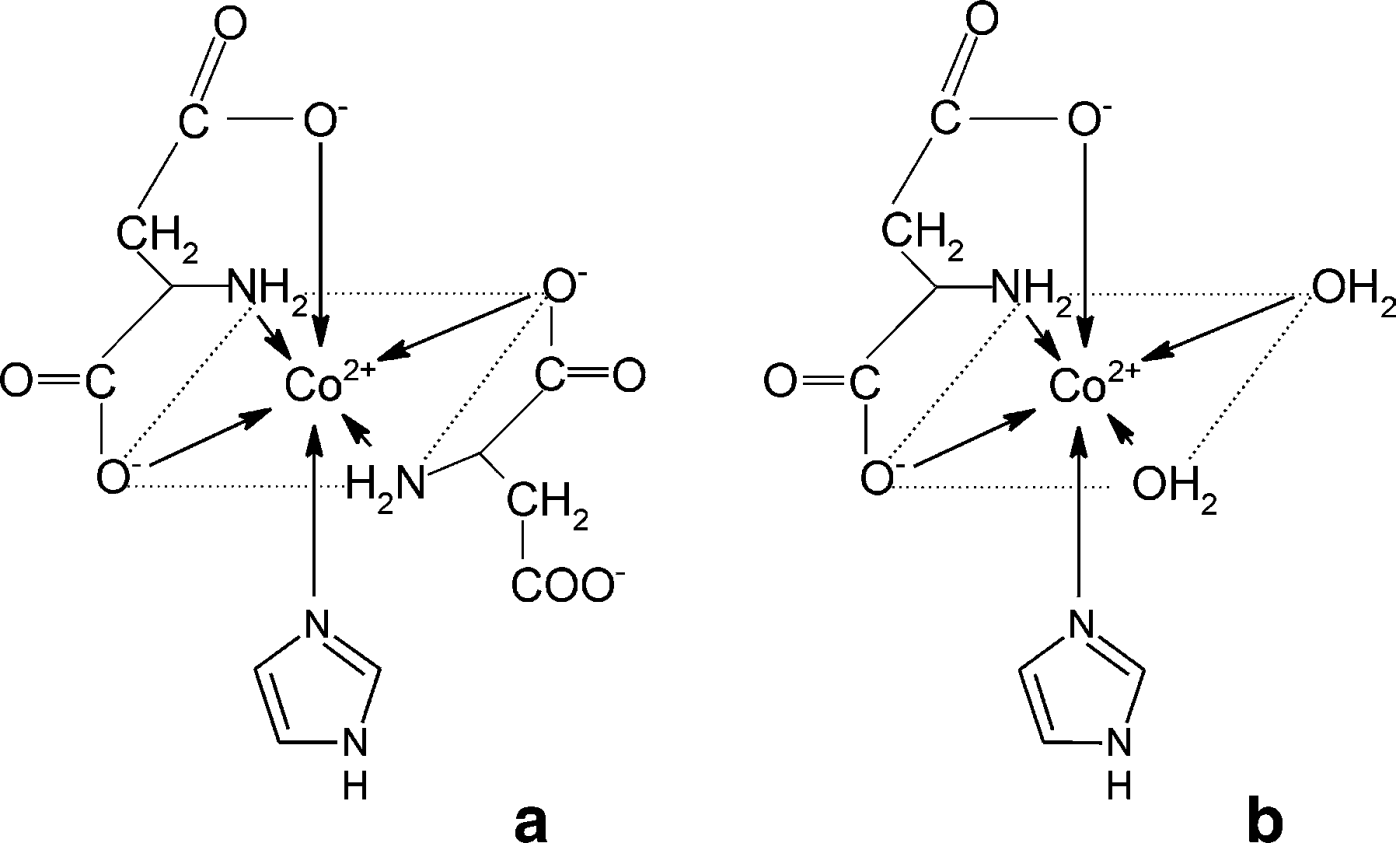
Suggested coordination modes of the ternary Co(II)–Himid–L-α-Asp complexes: **a** ML_2_L′; **b** MLL′

**Fig. 5:**
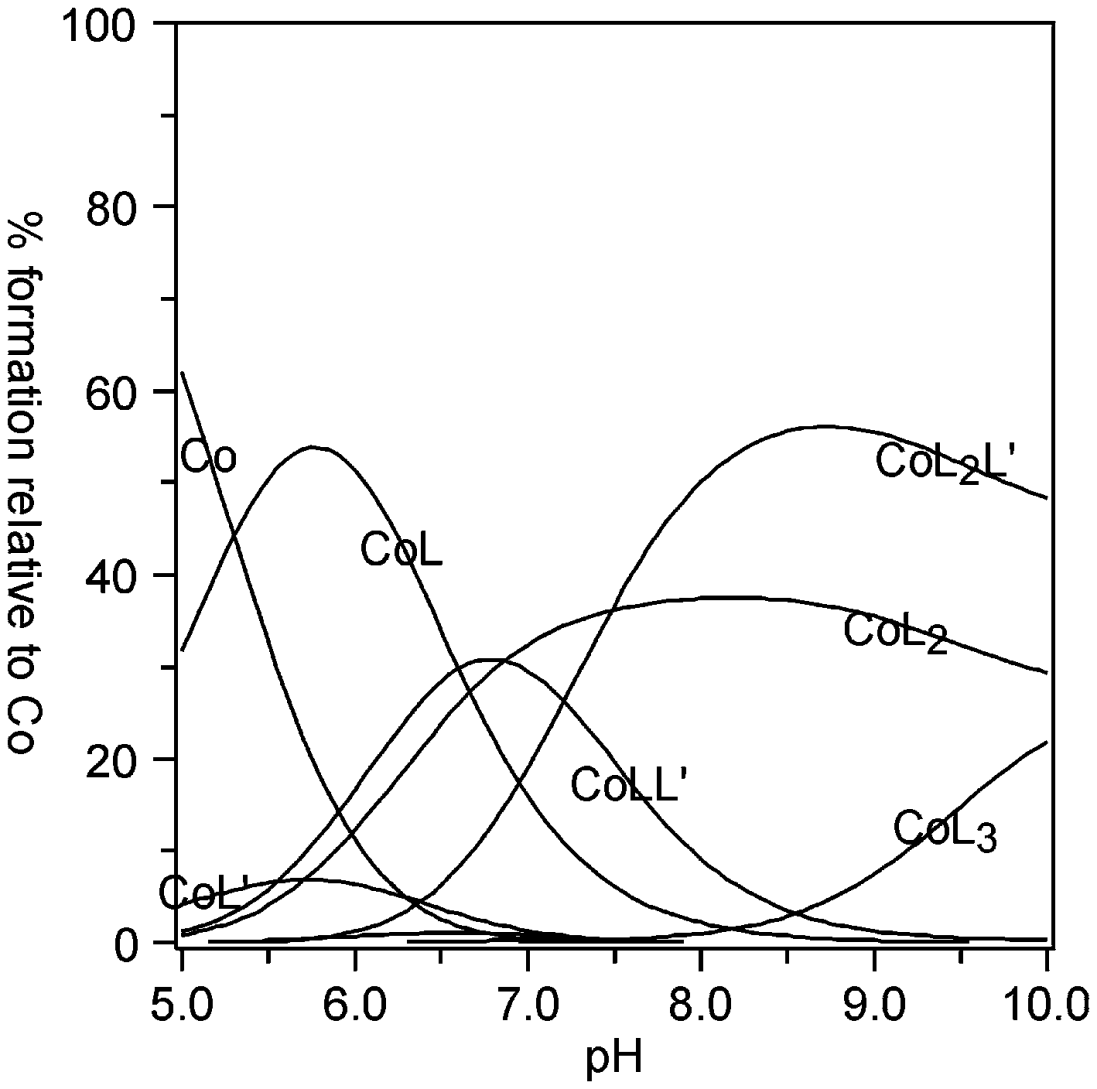
Distribution diagram of complex species versus pH for a solution of Co[(imid)_2_]_n_ and aspartic acid in molar ratio 1:5. *C*_Co_ = 0.01 mol L^−1^. L–aspartic acid (AspH_-1_), L′–imidazole (Himid)

As it follows from the speciation in Fig. 5, the ML_2_L′ heteroligand complex with aspartic acid (Fig. 4a) predominates in basic medium (pH > 7). It may be suggested that in this case, one of the amino acid molecules coordinates the metal via two carboxyl groups and one amino group (in place of equatorial and axial H_2_O). However, the second L molecule forms chelates only via α-COO^−^ and −NH_2_. The remaining carboxyl side group is not able to substitute imidazole from the opposite axial position due to the presence of a much weaker electron-pair donation than the imidazole N3 [16]. In turn, although only one amino acid molecule is involved in the formation of coordinative bonds in the MLL′ ternary complex (Fig. 4b), in this case, donation occurs via all the potential donors: α-COOH, β-COO^−^ and α-NH_2_. As can be seen in the speciation diagram (Fig. 5), the MLL′ complex exists in ca 30 % at pH 6.5–7.0.

### L-α-Lysine (Lys)

**Fig. 6:**
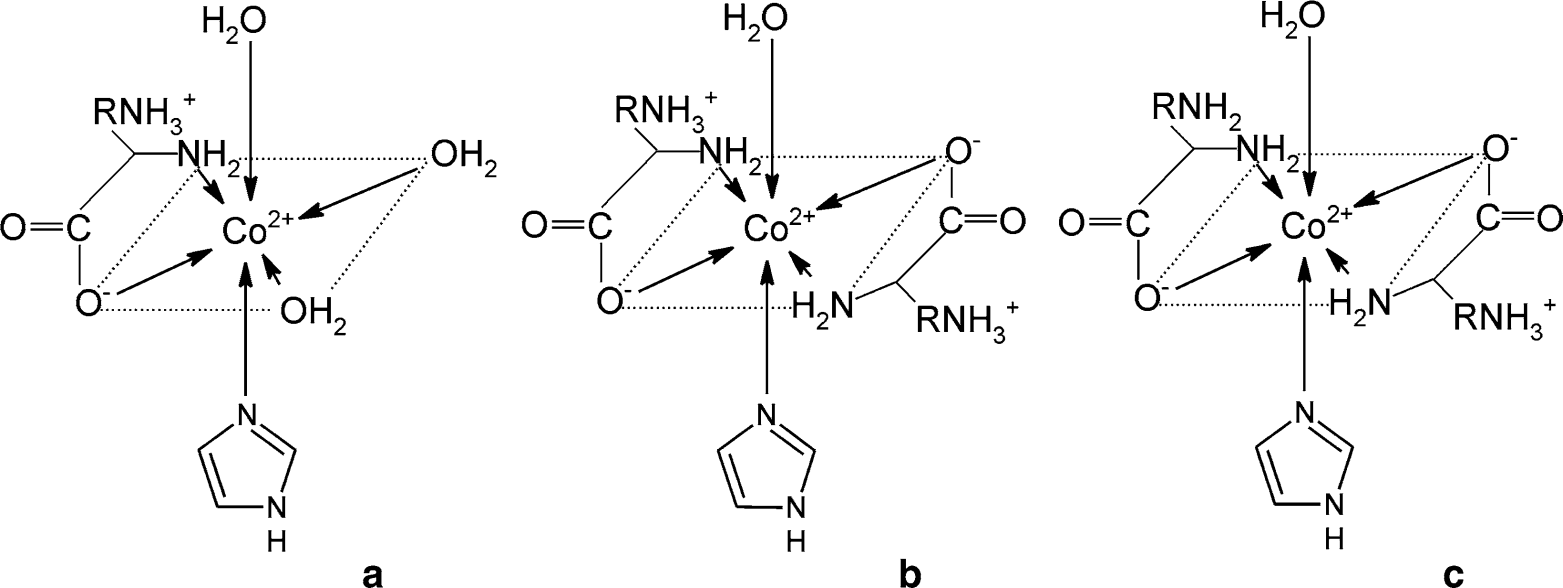
Suggested coordination modes of the ternary Co(II)–Himid–l-α-Lys complexes: **a** MLL′H; **b** ML_2_L′H_2_; **c** ML_2_L′H. R = (CH_2_)_4_

Three types of heteroligand complexes (MLL′H, ML_2_L′H, ML_2_L′H_2_) were confirmed in the lysine-containing systems. At higher pH values, the equilibrium set comprises a share of species with an amino acid molecule deprotonated at ε-NH_2_, owing to the proximity of the protonation constant of ε-NH_2_: 11.12 in logarithm (Table 1) and similar IUPAC data under analogous conditions [17]. Thus, the refinement results make it possible to propose three coordination modes (Fig. 6).

**Fig. 7:**
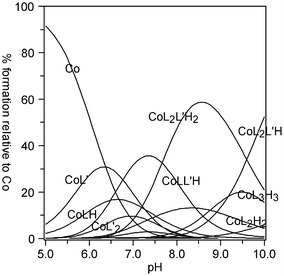
Distribution diagram of complex species versus pH for a solution of Co[(imid)_2_]_n_ and lysine in molar ratio 1:5. *C*_Co_ = 0.01 mol L^−1^. L–lysine (LysH_-1_), L**′**–imidazole (Himid)

In all of the species, lysine forms dative bonds with the central ion in the equatorial plane: via–COO^−^ and α-NH_2_. The complexes arise along with deprotonation of ε-NH_3_^+^ (Fig. 6) but this group is not likely to coordinate because an eight-membered ring at the axial position would be an unstable structure. The formation constant of ML_2_L′H_2_ becomes very high (Table 1), and its share in solution (up to 60 %) is the highest within the measurable pH range (Fig. 7).

### L-α-Histidine (His)

**Fig. 8:**
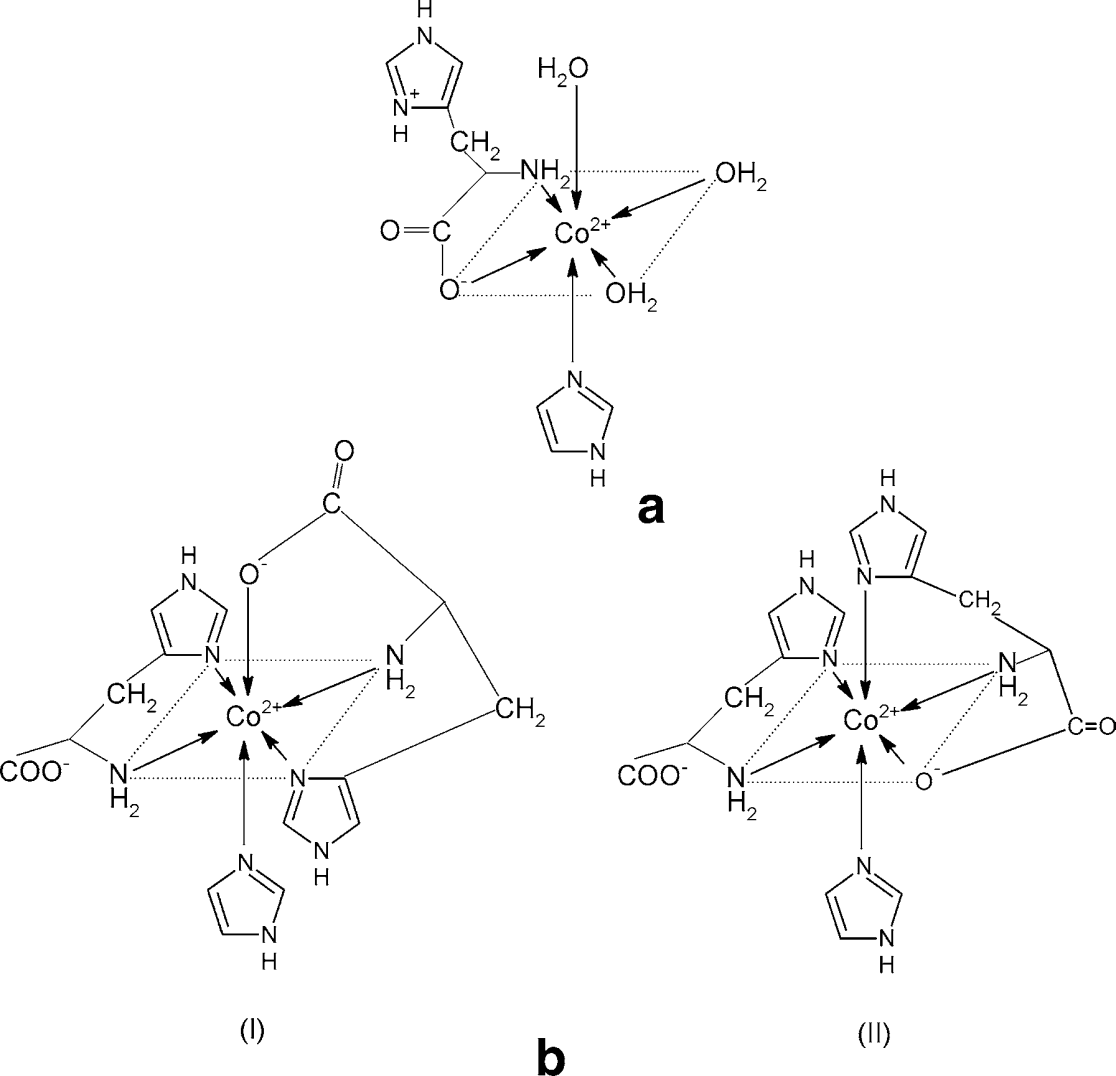
Suggested coordination modes of the ternary Co(II)–Himid–L-α-His complexes: **a** MLL′H; **b** ML_2_L′ (in two isomeric forms, I and II)

**Fig. 9:**
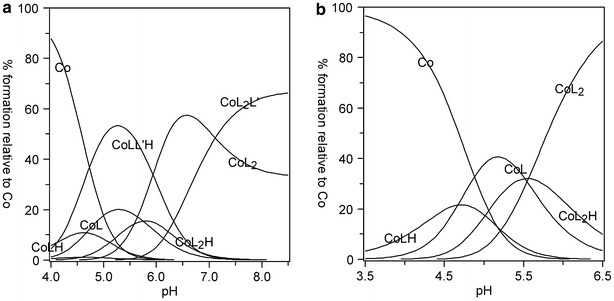
Distribution diagram of complex species versus pH for a solution of: **a** Co[(imid)_2_]_n_ and histidine in molar ratio 1:5. *C*_Co_ = 0.01 mol L^−1^. L–histidine (HisH_-1_), L′–imidazole (Himid); **b** Co(NO_3_)_2_ and histidine in molar ratio 1:5. *C*_Co_ = 0.04 mol L^−1^. L–histidine (His^−^)

In the cobalt(II)–histidine–imidazole system, both experimental methods confirmed the presence of two heteroligand species: MLL′H and ML_2_L′ (Fig. 8). Histidine is a potentially tetradentate ligand but in the measurable pH range, the imidazole N1 proton (p*K* 14.29) does not dissociate [18]. It follows from the speciation diagram that the MLL′H complex is formed within pH 4–7 (Fig. 9a). As it has been already suggested by literature CD data [2], at this pH range, histidine contains a protonated imidazole ring, whereas dissociation occurs at the carboxyl and amine groups. These groups take up two of the equatorial sites; the remaining three positions (two equatorial and one axial) are occupied by the solvent molecules H_2_O (as in Fig. 8a). In the ML_2_L′ complex, predominating at pH > 7, the histidine imidazole N3 undergoes deprotonation. Numerous potentiometric, calorimetric and spectroscopic studies [2] carried out for the binary ML_2_ cobalt(II)–histidine system have indicated that this complex occurs in solution in the form of an isomer mixture. Hence, analogous to our ML_2_L′ heteroligand complex, there is a possibility of amine and imidazole nitrogen atoms being coordinated in the equatorial positions. Thus, the –COO^−^ group of one of the histidines may be found in the axial position (Fig. 8b-I). Another probable form of this complex may occur also when a strongly dative imidazole N3 is coordinated in the axial position, substituting the H_2_O molecule, and then the –COO^−^ group moves to the equatorial site (Fig. 8b-II). The resulting species distribution (Fig. 9a) indicates a higher maximum share of ML_2_L′ than the protonated MLL′H complex, predominating in the more acidic medium.

**Fig. 10:**
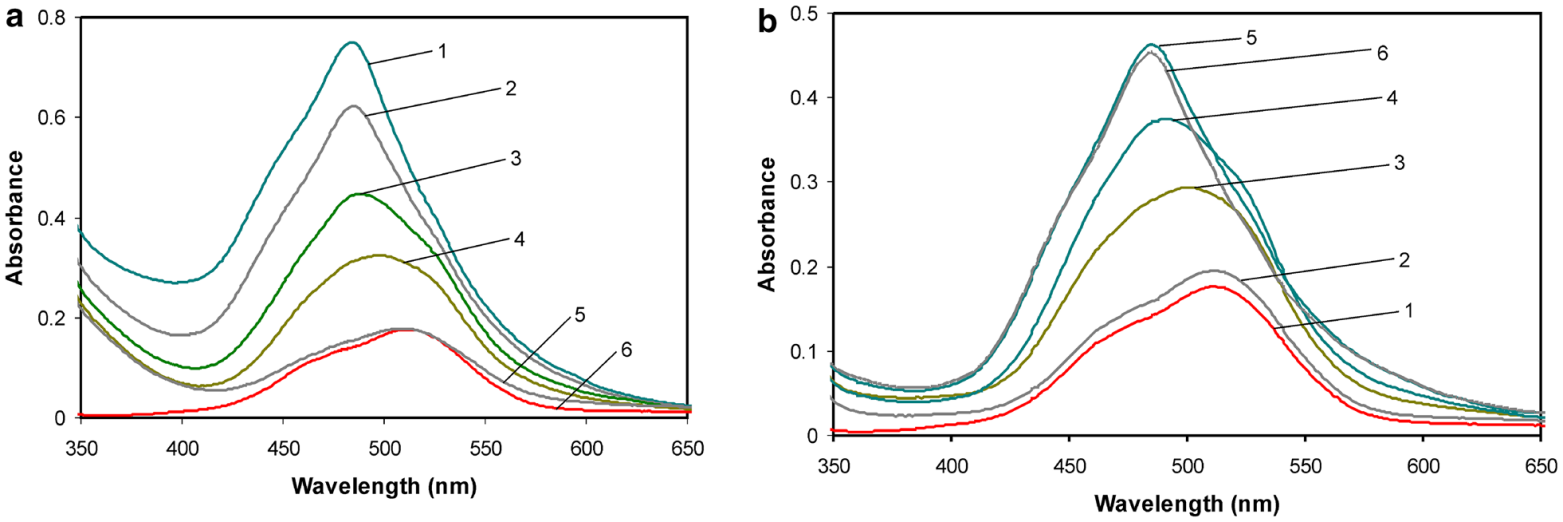
Vis absorption spectra of: **a** the heteroligand system in a solution containing [Co(imid)_2_]_n_ and histidine at 1:5 molar ratio (starting from basic solution of pH 8.64; *curve* 1). *C*_Co_ = 3.5 × 10^−2^ mol L^−1^. *Curves* 2–5 denote the spectra scanned after adding a consecutive portion of acid. pH: 2–6.15; 3–5.09; 4–4.92; 5–4.20. *Curve* 6—absorption spectrum of the Co(II) aquo-ion; **b** the binary system in a solution containing Co(II) and histidine at 1:5 molar ratio (starting from acid solution of pH 3.54; *curve* 2). *C*_Co_ = 3.5 × 10^−2^ mol L^−1^. *Curves* 3–6 denote the spectra scanned after adding a consecutive portion of base. pH: 3–4.61; 4–5.05; 5–6.12; 6–precipitate. *Curve* 1—absorption spectrum of the Co(II) aquo-ion

The visible absorption spectra, presented by way of example for cobalt(II)–histidine–imidazole (Fig. 10a), show stepwise dissociation of the heteroligand system to binary complexes which can be attributed to acidification. Finally, the binary complexes decompose to the cobalt(II) aqua-ion of *λ*_max_ = 512 nm (*ε* = 4.9), similar to our previous results for l-alanine [8]. For comparison, the literature data [19] referring to Co(H_2_O)_6_^2+^ are as follows: 515 nm (*ε* = 4.6). This band corresponds to a ligand field *d*–*d* transition T_1g_(F) → ^4^T_1g_(P) in admixture with a shoulder around 475 nm caused by spin forbidden transitions to doublet states. The hypsochromic shift becomes visible when comparing the spectra at higher and lower pH as a result of an exchange of the weaker *σ* donor (water) to much stronger function groups of the amino acids. Since the molar absorbance coefficients of binary complexes of cobalt with amino acids or imidazole are needed to study the equilibria with heteroligand complexes by Vis, they had to be determined independently prior to the calculations with the heteroligand species. Example absorption spectra of the binary Co(II)–histidine system are shown in Fig. 10b. The complexes of cobalt(II) and the ligand are formed along with alkalization starting from pH ca 3.5. Titration was carried out only to pH 6.12 due to precipitation following hydrolysis of the aqua-ion. It is important to note that the use of [Co(imid)_2_]_n_ as the starting compound, as described before, allows heteroligand complexes existing high above pH 6 to be created (cf. speciation in Fig. 9a) and to obtain relevant absorption spectra at pH 8.64 (Fig. 10a). A comparison of the two spectrophotometric titrations shows the differences within pH 5–6 as a result of higher share of the CoL and CoLH species of weaker ligand field power in the absence of imidazole (cf. Fig. 10b, higher shoulders of curves 3 and 4 at ca 530 nm). When comparing the speciations of Fig. 9a, b, it can be seen that the share of CoL is ca 40 % and the share of CoL_2_ up to 90 % in the absence of imidazole, whereas in the titrations with [Co(imid)_2_]_n_ the respective values are 20 and 60 %. Importantly, the absorption spectrum of the free Co(II) aqua-ion show the almost exact shape in Fig. 10a, b, which indicates lack of Co(II) oxidation to Co(III) during the experiments.

### Comparison of the stability constants of the heteroligand complexes in the Co^II^(Himid)(L-α-Amac)_n_ system

It is essential to compare the values of log_10_ *β*_*mll′h*_ stability constants of the heteroligand complexes Co(II)(Amac)_2_(Himid), which are potential models of dioxygen carriers in solution. Assuming that one of them (Lys) contains the Amac ligand in a protonated AmacH or AmacH_2_ form, it was necessary in this case to subtract the protonation constant log_10_ *β*_0101_ or log_10_ *β*_0102_ from log_10_ *β*_1211_ or log_10_ *β*_1212_, respectively. Finally, it may be concluded from Table 1 that the comparable, corresponding stability constants of Co(II)(Amac)_2_(Himid) follow the series: His > Asp > Lys > Ala > Asn = 14.61 > 12.24 > 11.45 (11.65) > 9.94 > 9.70. It may be suggested that the effect of the stacking interaction between the aromatic ring of amino acid and imidazole in the Co^II^(Himid)(His) is responsible for the highest value among all of the Amac ligands. On the other hand, Co^II^ (Asp)_2_(Himid) is the most favored heteroligand species from the ones with aliphatic side chains, most probably due to coordination of the carboxyl oxygen *trans* to the axial imidazole N3.

Moreover, there are different methods allowing the stability of heteroligand complexes to be compared with those of the corresponding binary systems. One such method is calculation of the stabilization constant (Δlog_10_*β*) [11, 20] (Eq. 3) on the grounds of the difference between the experimental stability constant for the mixed-ligand complex (log_10_*β*_1110_) and the constant based on statistical data (log_10_*β*_stat_):

3 $$\Delta { \log }_{ 10} \beta = { \log }_{ 10} \beta_{ 1 1 10} - { \log }_{ 10} \beta_{\text{stat}}$$

where:

4 $${ \log }_{ 10} \beta_{\text{stat}} = { \log }_{ 10} 2 { } + \, \left( { 1/ 2} \right){ \log }_{ 10} \beta_{ 1 200} + \, \left( { 1/ 2} \right){ \log }_{ 10} \beta_{ 1 0 2 0} .$$

For lysine and histidine, heteroligand complexes with one amino acid which was always protonated were identified (Table 1). Therefore, Δlog_10_*β* and log_10_*β*_stat_ were calculated on the basis of Eqs. (5), (6):

5 $$\Delta { \log }_{ 10} \beta = { \log }_{ 10} \beta_{ 1 1 1 1} - { \log }_{ 10} \beta_{\text{stat}}$$

6 $${ \log }_{ 10} \beta_{\text{stat}} = { \log }_{ 10} 2 { } + \, \left( { 1/ 2} \right){ \log }_{ 10} \beta_{ 1 20 2} + \, \left( { 1/ 2} \right){ \log }_{ 10} \beta_{ 10 20} .$$

Table 2 presents the values of the stabilization constants for the four heteroligand complexes. Δlog_10_ *β* was not calculated for histidine because the stability constant of the binary complex containing two protonated amino acids (Table 1) is unavailable. The large differences between experimental and calculated stability constants Δlog_10_*β* indicate that the heteroligand species are more stable than the binary ones. The heteroligand complex with aspartic acid has the highest value of Δlog_10_*β*, suggesting that formation of the binary complex involving two molecules of the tridentate ligand (juxtaposed to the binary species with bidentate alanine, asparagine and protonated lysine) is less favoured than the heteroligand complex with one tridentate ligand. This may be easily explained by that the initial Co^II^(Himid) moiety has even five available coordination sites that can be occupied by two carboxyl groups and one amino group.

**Table 2:** Evaluated values of Δlog_10_ *β*, Δlog_10_ *K*, log_10_ *X* used for comparison of the stability of the heteroligand Co^II^(Himid)(L-α-Amac)_n_ complexes with their parent binary complexes

Ligand	log_10_ *β*_1110_ (experimental)	log_10_ *β*_stat_^a^ (calculated)	Δlog_10_ *β* ^b^	Δlog_10_ *K* ^c^	log_10_ *X* ^d^
Alanine	6.97	6.60	0.37	−0.05	1.35
Asparagine	6.89	6.61	0.28	−0.18	1.17
Aspartic acid	8.30	7.85	0.45	−0.24	1.50
Lysine	17.78^e^	17.43^f^	0.35^g^	−0.08^h^	1.30^i^
Histidine	16.30^e^	–^f^	–	2.07^h^	–^i^

^a^ log_10_ *β*_stat_ = log_10_ 2 + (1/2)log_10_ *β*_1200_ + (1/2) log_10_ *β*_1020_

^b^ Δlog_10_ *β* = log_10_ *β*_1110_−log_10_ *β*_stat_

^c^ Δlog_10_ *K* = log_10_ *β*_1110_−log_10_ *β*_1010_−log_10_ *β*_1100_

^d^ log_10_ *X* = (2 log_10_ *β*_1110_−log_10_ *β*_1200_−log_10_ *β*_1020_)

^e^ For log_10_ *β*_1111_

^f^ log_10_ *β*_stat_ = log_10_ 2 + (1/2)log_10_ *β*_1202_ + (1/2) log_10_ *β*_1020_

^g^ Δlog_10_ *β* = log_10_ *β*_1111_−log_10_ *β*_stat_

^h^ Δlog_10_ *K* = log_10_ *β*_1111_−log_10_ *β*_1010_−log_10_ *β*_1101_

^i^ log_10_ *X* = (2 log_10_ *β*_1111_−log_10_ *β*_1202_−log_10_ *β*_1020_)

Another very important parameter used to compare the stabilization of the heteroligand complexes with their binary system is Δlog_10_*K* [21]. It is calculated according to Eq. (7) as the difference between the stability constants for the deprotonated mixed-ligand, Co^II^(Himid)(AmacH_−1_) and two binary, Co^II^(Himid) and Co^II^(AmacH_−1_), complexes:

7 $$\Delta { \log }_{ 10} K = { \log }_{ 10} \beta_{ 1 1 10} - { \log }_{ 10} \beta_{ 10 10} - { \log }_{ 10} \beta_{ 1 100}$$

For the complexes containing protonated ligand forms (lysine and histidine), Δlog_10_*K* is calculated as shown in the Eq. (8) [11]:

8 $$\Delta { \log }_{ 10} K = { \log }_{ 10} \beta_{ 1 1 1 1} - { \log }_{ 10} \beta_{ 10 10} - { \log }_{ 10} \beta_{ 1 10 1}$$

The parameter Δlog_10_*K* describes the influence of the bonded primary ligand in the binary complex Co^II^(Himid) towards an incoming secondary ligand (L) forming a heteroligand complex. The negative values (Table 2) indicate that the mixed-ligand systems are less stable than the binary complexes with one molecule of imidazole or one molecule of amino acid, in contrast to Δlog_10_ *β*, which deals with binary complexes Co^II^(Himid)_2_ and Co^II^(AmacH_−1_)_2_ containing two ligand molecules. More coordination positions are available for bonding the first ligand than the second ligand [21]. An exception is the positive value of Δlog_10_*K* for the heteroligand complex MLL′H with histidine (Table 2). By comparing the structure of this ligand with that of other amino acids, it can be seen that histidine has an aromatic ring containing N as a donor atom, which affects the stability of the heteroligand complex [21, 22]. Similar aromatic ring stacking has been observed in mixed-ligand complexes formed by two different ligands which contain aromatic rings [23]. At least one of these rings has to be incorporated in a flexible side chain, just as it occurs in the histidine containing MLL′H species. The other ring may also be of the flexible type or it may be rigidly fixed to the metal ion, as is the case with imidazole. Evidently, a stacking interaction occurring between aromatic ring of amino acid and imidazole in the Co^II^(Himid)(L-α-Amac) system leads to a higher stability of this heteroligand complex than the binary complex with one protonated histidine. The intramolecular ligand–ligand interaction may also be possible between the aliphatic chain of the amino acid and aromatic ring of the second ligand. Qualitative observations found that the extent of the intramolecular interaction in the complexes increases in the following series: aliphatic–aliphatic < aliphatic–aromatic < aromatic–aromatic [24]. This accounts for the lower stability of the mixed-ligand complexes containing aliphatic amino acids (negative value of Δlog_10_*K*) in comparison with the heteroligand complex of the histidine.

Another parameter which enables the stability of the mixed-ligand complexes to be determined is the disproportionation constant log_10_*X* (Table 2) [11]. Like Δlog_10_*β*, log_10_*X* is based on the stability constants of the binary complexes with two ligand molecules (Eq. (9) for deprotonated amino acids and Eq. (10) for protonated lysine):

9 $${ \log }_{ 10} X = \, ( 2 {\text{log}}_{ 10} \beta_{ 1 1 10} - { \log }_{ 10} \beta_{ 1 200} - { \log }_{ 10} \beta_{ 10 20} )$$

10 $${ \log }_{ 10} X = \, ( 2 {\text{log}}_{ 10} \beta_{ 1 1 1 1} - { \log }_{ 10} \beta_{ 1 20 2} - { \log }_{ 10} \beta_{ 10 20} )$$

Higher values of log_10_*X* indicate more stable heteroligand complexes than their binary counterparts. Comparison of the calculated data log_10_*X* with Δlog_10_*β* values leads to the same conclusion.

## Conclusions

The experimental results make it possible to conclude that mixed-ligand complexes of MLL′ type are present in the equilibrium mixture created by [Co(imid)_2_]_n_ and Amac already at pH >4–6. On the other hand, the heteroligand ML_2_L′ species, known as the “active complex”, as it is able to take up dioxygen in a reversible manner, predominate within the higher pH range and attain their maximum share at pH ~9. Our present findings allow the oxygenation constants to be evaluated in full accordance with the species distribution in solution. Such calculations are required also in our laboratory as part of investigations of artificial blood-substituting systems. By knowing the formation constants of the heteroligand ML_2_L′ species, it is possible to compare their stability in solution. For the group of amino acids used in the present work, the highest value of stability constant was found for L-α-histidine with a heterocyclic side ring, which leads to relative high concentration of the “active” ML_2_L′ species in the equilibrium mixture. From among the other Amac ligands with aliphatic side groups, the highest stability constant of ML_2_L′ was evidenced for L-α-aspartic acid.

Various methods allow a comparison of the stability of heteroligand complexes with that of the corresponding binary systems. The stabilization constant Δlog_10_*β* calculated on the basis of the difference between the experimental stability constant for the mixed-ligand complex MLL′ (log_10_*β*_1110_) and the constant based on statistical data (log_10_*β*_stat_) indicate that the heteroligand species are more stable than the binary ones. The parameter Δlog_10_*K*, used to compare the stabilization of the heteroligand complexes with their binary system by showing the difference between the stability constants for the deprotonated mixed-ligand, Co^II^(Himid)(AmacH_−1_), and two binary complexes, Co^II^(Himid) and Co^II^(AmacH_−1_), demonstrates the lower stability of the mixed-ligand complexes containing aliphatic amino acids (negative value of Δlog_10_*K*) in comparison with the heteroligand complex of the histidine. Also, both the disproportionation constant log_10_*X* and the Δlog_10_*β* value indicate that the heteroligand complexes are more stable than their binary counterparts.

Furthermore, it is evident from our studies that excess of amino acid in solution prior to reaction results in an increasing percentage of the Co^II^(Himid)(Amac)_2_ heteroligand species as compared with Co^II^(Himid)(Amac). The use of amino acid in excess leads also to a rise in the share of the binary cobalt(II)–amino acid compounds together with a decreasing share of binary cobalt(II)–imidazole complexes. For all the amino acids involved, greater excesses of amino acid are associated with lower pH values for which the heteroligand complex reaches maximum share.

## Experimental

### Reagents

The procedure for preparation of the polymeric, pseudo-tetrahedral, semi-conductive Co(II) complex with imidazole–[Co(imid)_2_]_n_, as well as analytical and IR spectroscopic identification, have already been reported in [3, 4, 8]. The purity of amino acids used in this investigation: L-α-asparagine (Sigma Aldrich), L-α-aspartic acid (Fluka), L-α-lysine (Sigma Aldrich) and L-α-histidine (Fluka) was determined potentiometrically. Imidazole p.a. was purchased from Merck. Alkali (0.5 mol L^−1^ NaOH, carbonate-free) was purchased from Malinckrodt Baker B. V. Cobalt(II) nitrate hexahydrate, potassium nitrate, nitric acid (POCh Gliwice) were also p.a. reagents. Argon (99.999 %) from Linde Gas (Poland) was used.

### General potentiometric procedures

The protonation constants of ligands and formation constants of binary complexes was determined by means of a MOLSPIN automatic titration kit (Molspin Ltd, Newcastle-upon-Tyne). A Hamilton Bonaduz AG microsyringe for 250 μL was used with the auto burette. The titrant (0.5 mol L^−1^ NaOH) was taken from an external flask protected from CO_2_. The measurements were carried out with a combined OSH 10–10 electrode (Metron, Gliwice) in a thermostated vessel at initial volume 4.00 mL, constant temperature 25.0 ± 0.1 °C and ionic strength *I* = 0.5 mol L^−1^ (KNO_3_). Prior to the proper titrations, a two-buffer standardization of the electrode according to [25] was performed, and the measurement cell was then calibrated in the EMF = −log_10_ [H^+^] scale by strong acid–strong base titration at the same constant temperature and constant ionic strength *I* = 0.5 mol L^−1^ (KNO_3_) according to [26]. The values of standard electromotive force, *E*_0_ (which also takes into account the liquid junction potential) and slope, *s* from equation $E = E_{0} - s\, \cdot 59.16 \cdot \;( - \log_{10} \,[{\text{H}}^{ + } ])$ were then subsequently inserted in the input files used to evaluate the overall, concentration formation constants.

### Potentiometric procedures in protonation of the amino acids and imidazole

The titrations of amino acids and imidazole solutions were carried out under the same conditions as in the calibration procedures. Nitric acid was used as a mineral acid added prior to titrations. The following acidified ligand solutions of three various concentrations were used—asparagine: (1.5; 1.75; 2.0) × 10^−2^ mol L^−1^ at ligand to mineral acid molar ratio 1: 0.9, aspartic acid: (1.0; 1.1; 1.2) × 10^−2^ mol L^−1^ at ligand to mineral acid molar ratio 1:1.2, lysine: (1.0; 1.1; 1.2) × 10^−2^ mol L^−1^ at ligand to mineral acid molar ratio 1:2, histidine: (1.0; 1.1; 1.2) × 10^−2^ mol L^−1^ at ligand to mineral acid molar ratio 1:2, imidazole: (1.9; 2.0; 2.1) × 10^−2^ mol L^−1^ at ligand to mineral acid molar ratio 1.5:2.

### Potentiometric procedures in determination of Co^II^(Himid)_n_ and Co^II^(L-α-Amac)_n_ complexing equilibria under oxygen-free conditions

Solutions containing metal and ligand were prepared at three molar L:M ratios (from 2: 1 to 5:1), with exception of lysine (from 3:1 to 7:1) and imidazole (from 5:1 to 10:1). During preparation, the solutions, initially in absence of cobalt, were acidified to a various extent of mineral acid to ligand: asparagine and aspartic acid molar ratio 0.1:1; lysine and histidine 1:1; imidazole 1.1:1. The titrations were carried out under pure argon.

### Potentiometric procedures in determination of heteroligand oxygen-free cobalt(II)–L-α- amino acid–imidazole complexes under oxygen-free conditions

**Fig. 11:**
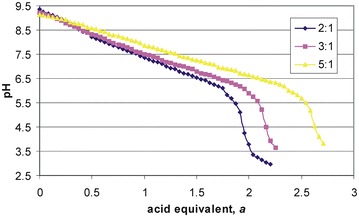
Representative titrations of the [Co(imid)_2_]_n_–L-α-lysine system under argon. *C*_Co_ = 0.01 mol L^−1^. *Curves* correspond to various amino acid-to-cobalt ratios

An isobaric laboratory set was used for pH-metric and volumetric measurements. Prior to each experiment, the glass electrode was standardized with two buffers (pH 4.00 and pH 9.00, Russell pH Ltd) at 25.0 ± 0.1 °C. The thermostated measurement vessel with an aqueous solution of initial volume 30.0 mL contained the amino acid and potassium nitrate to maintain ionic strength *I* = 0.5 mol L^−1^. Prior to the experiments, the solutions of aspartic acid and histidine were alkalized with 0.5 mol L^−1^ NaOH (molar ratio aspartic acid to base 1:1.2 and histidine to base 1:0.25), whereas the solution of lysine was acidified with 0.5 mol L^−1^ HNO_3_ (molar ratio ligand to mineral acid 1:0.5). The initial pH measured with a PHM 85 precision pH-meter Radiometer (Copenhagen) and combined C2401 electrode ranged within 5–6. A small glass vessel with 0.3 mol of the polymeric [Co(imid)_2_]_n_ complex (i.e. 0.3 mol of Co and 0.6 mol of imidazole) was hung from a glass rod over the solution surface and then the entity was tightly closed with a silicon stopper and purged with pure argon. During continuous gas flow and constant stirring, the polymer was inserted into the sample and then, after dissolution, each Co(imid)_2_ unit could form complexes with the amino acid. As a result of the evolution of one imidazole molecule into solution from each Co(imid)_2_ unit, the pH rose to about 9–10. Finally, three solutions of three molar L:M (2:1, 3:1, 5:1 at constant amount of [Co(imid)_2_]_n_) were titrated with 0.5 mol L^−1^ HNO_3_ until pH decreased to ca 4. A change in color from deep-violet to pink was observed during the titration. The titration end point (*a*= 2) corresponded to neutralization of the total content of 0.6 mol imidazole involved in the Co(imid)_2_ unit of the polymeric [Co(imid)_2_]_n_ complex (Fig. 11). At higher excess of the amino acids containing a third functional group (e.g. for lysine), the end point moved towards higher values.

### Spectrophotometric procedures in the determination of Co^II^(Himid)_n_ and Co^II^(L-α-Amac)_n_ complexing equilibria under oxygen-free conditions

The experiments were carried out by means of a Cary 50 Bio UV–Visible spectrophotometer, slit width 1.5 nm (Varian Pty. Ltd., Australia) equipped with Peltier accessory (temp. 25.0 ± 0.1 °C). Acidified (HNO_3_) solutions of the ligands and Co(NO_3_)_2_ at ionic strength *I* = 0.5 mol L^−1^ (KNO_3_) were prepared in three molar ratios L:M, corresponding to the potentiometric measurements described before. All the investigations were made within concentration range of Co(NO_3_)_2_ (3.0–6.5) × 10^−2^ mol L^−1^. The solution of initial volume 2.40 mL was placed in a weighted empty cell, closed by a silicon stopper. Then, after argonation for 15–20 min, the solution was titrated by small aliquots (0.10–0.20 mL) of de-aerated 0.5 mol L^−1^ NaOH of known density, up to a precipitation caused by hydrolysis of the Co(II) aqua-ion at higher pH [29]. After each aliquot of alkali, the cell was weighed before recording the UV/Vis spectrum.

### Spectrophotometric studies of the dissociation of the heteroligand cobalt(II)–L-α-amino acid–imidazole complexes

Solutions were prepared with a Co(imid)_2_ to amino acid molar ratio of 1:2, 1:3 or 1:5. The total concentration of cobalt amounted to 3.5 × 10^−2^ mol L^−1^. Appropriate amounts of amino acid, polymer and potassium nitrate (*I* = 0.5 mol L^−1^) were weighed directly in a silica cell. The cell was closed with a silicon stopper, rinsed with pure argon and then the necessary volume of argonated water was added to make the sample up to 2.40 mL. Initial solutions of some amino acids were alkalized with 0.5 mol L^−1^ NaOH (aspartic acid and histidine) or acidified with 0.5 mol L^−1^ HNO_3_ (lysine), in order to attain an initial pH ~9. The solution inside the cell was then titrated with portions of argonated 0.5 mol L^−1^ HNO_3_ of known density. Each dose of the titrant amounted to 0.10–0.20 mL. The cell with a silicon stopper was weighed after each titration step and then the UV/Vis absorption curve was taken by the spectrophotometer at 25.0 ± 0.1 °C.

### Calculations

Following the potentiometric titrations of amino acids and imidazole in the absence of the metal, of amino acids and imidazole along with Co(II) and then of amino acids in presence of the [Co(imid)_2_]_n_ polymer, all the formation constants were determined consecutively using the Hyperquad 2008 fitting procedure [27] under the same temperature and medium. Goodness of fit was controlled by the objective function *U* = *Σ*_*i*=*1,m*_*W*_*i*_*r*_*i*_^*2*^, where *W* is the weight and *r* is the residual, equal to the difference between observed and calculated values of the electromotive force EMF (*E*_*exp*_–*E*_*theoret*_), *m*—number of experimental points, *n*—number of refined parameters. The weighting factor *W*_i_ is defined as the reciprocal of the estimated variance of measurements in dependence on the estimated variances of EMF and volume readings. The normalized sum of squared residuals, *σ* = *U/*(*m*−*n*) was compared with a *χ*^2^ (Chi squared) test of randomness at a number of degrees of freedom equal to *m*−*n*. Our value for the ionic product of water under the corresponding conditions, p*K*_w_ = 13.94, was in close accordance with the literature 13.97 [17]. For each system, data from different titrations was taken together in a comprehensive file. Graphical simulations of speciation diagrams on the basis of calculated constants were created using HySS 2009 [28]. The equilibrium models with potentiometrically-determined formation constants were consecutively confirmed spectrophotometrically by using HypSpec (part of Hyperquad suite, Protonic Software). The HypSpec program resolves a linear equation system based on Lambert–Beer’s law, using the spectrophotometric data and known (or estimated) equilibrium constants, yielding the molar extinction coefficients of individual absorbing species. Then, optionally the program can be used to refine the estimated equilibrium constants from spectrophotometric data.

## Abbreviations

Amac: amino acid
imid: imidazole
Asn: asparagine
Asp: aspartic acid
Lys: lysine
His: histidine
L = AmacH_-1_: amino acid ligand in fully deprotonated form, as commonly recognized in equilibrium studies
L′ = Himid: imidazole ligand found in the species of equilibrium mixture
M = Co^2+^: metal center
L:M: amino acid ligand-to-metal concentration ratio
*a*: mmole of base/mmole of ligand
*β*_*mll’h*_ = [M_*m*_L_*l*_L′_*l’*_H_*h*_]/[M]^*m*^[L]^*l*^[L′]^*l’*^[H]^*h*^: cumulative stability constant
*m*, *l*, *l*′, *h*: number of metals (central ions), ligands and protons, respectively concentrations in square brackets denote equilibrium concentrations
